# “Everything within a Circle Is One Thing”

**DOI:** 10.3201/eid2312.AC2312

**Published:** 2017-12

**Authors:** Byron Breedlove

**Affiliations:** Centers for Disease Control and Prevention, Atlanta, Georgia, USA

**Keywords:** about the cover, art science connection, art and medicine, emerging infectious diseases, Everything within a Circle Is One Thing, A Timeless Symbiosis, Bindu Viswanathan, zoonosis, One Health

**Figure:**
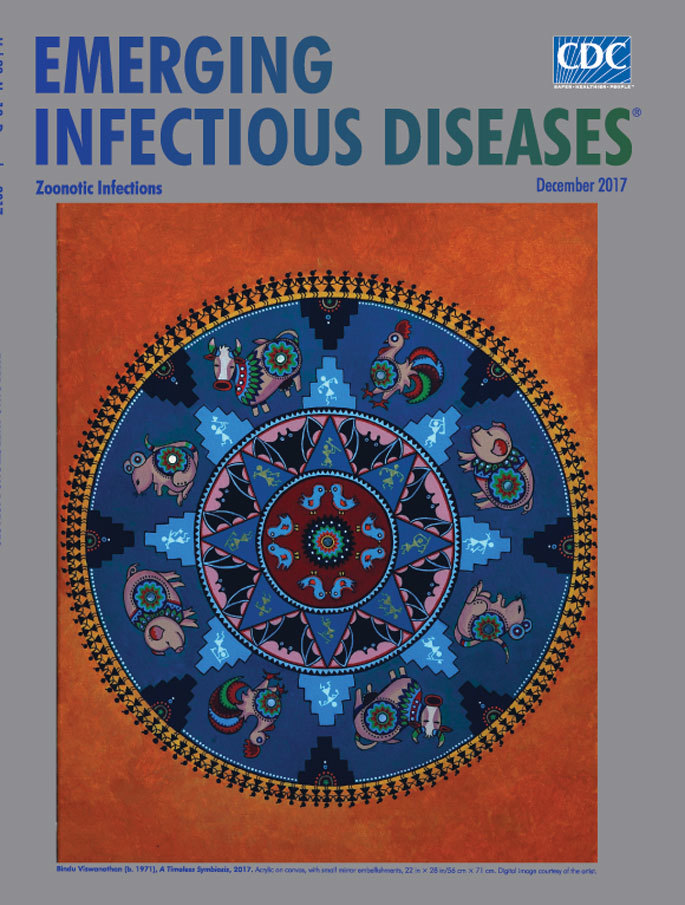
**Bindu Viswanathan, (b. 1971), A Timeless Symbiosis, 2017.** Acrylic on canvas, with small mirror embellishments, 22 in × 28 in/56 cm × 71 cm. Digital image courtesy of the artist.

“Everything within a circle is one thing, which is encircled, enframed,” wrote 20^th^-century American mythologist and lecturer Joseph Campbell. This month’s cover art, *A Timeless Symbiosis,* uses the motif of circles to connect animals and humans. Bindu Viswanathan, the artist who created the painting, is also a lecturer and biostatistician at the University of Texas, Austin. Many of her paintings inspire viewers to think about the fuller connection between animals and humans. According to Viswanathan, “Circles have no beginning and no end, much like the interdependence of humans and animals. They also represent the womb, the origin of life, and its continuum.” (All quotes from B. Viswanathan, pers. comm. Nov 3, 2017.)

Viswanathan incorporates elements from the Indian tribal art of the Warli, an indigenous people who live in the hills of western India, into *A Timeless Symbiosis.* For instance, Warli art, which has been practiced since at least the 10th century ace, often depicts the intertwined links of humans with both domestic and wild animals.

Viswanathan’s meticulous interplay of geometric forms, vibrant colors, calculated symmetry, and traditional symbols is arrayed within concentric circles and draws the viewer deeper and deeper into the painting. The outer circular border comprises 122 Warli-style figures, linked hand in hand, keeping vigil. Within the next circle, against a dark blue background, ornamentally decorated pairs of pigs, cows, chickens, and rats—all animals associated with domestication—are interspersed among figures of humans, posed as though engaged in traditional agrarian tasks. The disproportionate size of the animals underscores their importance to and impact on the people.

More human figures are juxtaposed with bats and birds, wild animals many humans are likely to encounter and major sources of diverse zoonotic viruses throughout the world. The radiating central image contains what is perhaps best described as a viewer’s choice, possibly a sun or essential life force, or maybe a virus or other microscopic organism.

Throughout each of the circles, the artist incorporates various geometric forms—stair-stepped pyramids, triangles, and circles—and then reuses those forms to create ornate, complex designs. Viswanathan notes that “I gravitate toward geometric designs and symmetry in my abstract art, which stems from the same source that is inspired by mathematics. As a biostatistician, I model complex relationships that exist in the natural world.”

Once a viewer is drawn to the center of the painting, the tendency is to move back through the circles to the edges of the fiery orange border and then reexamine the careful symmetry, much like the process of viewing a mandala. The artist portrays her theme of symbiosis on multiple levels and illustrates the complex relationships and connections between humans and animals.

Rudolph Virchow, one of the 19th century’s principal leaders in medicine and pathology, stated that “Between animal and human medicine, there is no dividing line—nor should there be.” Virchow stressed that diseases of humans and animals are interconnected and devised the term “zoonosis” to describe the links between infectious diseases in animal and human health. The ring of people forming the outer edge of *A Timeless Symbiosis* can serve to symbolize the modern One Health concept, an initiative that requires close collaboration among researchers in the animal, human, and environmental health sectors to protect and preserve life.
